# The Response of the Soil Microbiota to Long-Term Mineral and Organic Nitrogen Fertilization is Stronger in the Bulk Soil than in the Rhizosphere

**DOI:** 10.3390/genes11040456

**Published:** 2020-04-22

**Authors:** Massimiliano Cardinale, Stefan Ratering, Aitak Sadeghi, Sushil Pokhrel, Bernd Honermeier, Sylvia Schnell

**Affiliations:** 1 Department of Biological and Environmental Sciences and Technologies, University of Salento, I-73100 Lecce, Italy; 2 Institute of Applied Microbiology, Justus-Liebig-University Giessen, D-35342 Giessen, Germany; stefan.ratering@umwelt.uni-giessen.de (S.R.); sylvia.schnell@umwelt.uni-giessen.de (S.S.); 3 Institute of Agronomy and Plant Breeding I, Justus-Liebig-University Giessen, D-35342 Giessen, Germany

**Keywords:** Long-Term Experiments (LTE), nitrogen fertilization, soil microbiota, nitrogen cycle, quantitative PCR, high-throughput sequencing, co-occurrence network

## Abstract

The effects of different agronomic practices, such as fertilization regimes, can be experimentally tested in long-term experiments (LTE). Here, we aimed to evaluate the effect of different nitrogen fertilizations on the bacterial microbiota in both rhizosphere and bulk soil of sugar beet, in the Giessen-LTE (Germany). Fertilization treatments included mineral-N, manure, mineral-N + manure and no N-amendment. Metabarcoding and co-occurrence analysis of 16S rRNA genes, qPCR of *amoA*, *nirK*, *nirS*, *nosZ*-I and *nosZ*-II genes and soil physico-chemical analyses were performed. The effect of the fertilization treatments was more evident in the bulk soil, involving 33.1% of the microbiota. Co-occurrence analysis showed a rhizosphere cluster, dominated by *Proteobacteria, Actinobacteria* and *Verrucomicrobia* (hub taxa: *Betaproteobacteriales*)*,* and a bulk soil cluster, dominated by *Acidobacteria*, *Gemmatominadetes* and “*Latescibacteria*” (hub taxa: *Acidobacteria*). In the bulk soil, mineral N-fertilization reduced *nirK*, *amoA*, *nosZ*-I and *nosZ*-II genes. Thirteen Operational taxonomic units (OTUs) showed 23 negative correlations with gene relative abundances. These OTUs likely represent opportunistic species that profited from the amended mineral-N and outgrew the species carrying N-cycle genes. Our results indicate trajectories for future research on soil microbiome in LTE and add new experimental evidence that will be helpful for sustainable management of nitrogen fertilizations on arable soils.

## 1. Introduction

Long-term experiments (LTE) in agriculture help to assess the effects of different agronomical practices and soil managements on plant growth, crop yields, ecosystem response and soil biological parameters [1,2]. LTE have been established in agricultural research stations worldwide. Different fertilization regimes (especially mineral *vs.* organic) have shown to optimize soil management, in order to reduce the input of chemicals into the soil [3,4,5] and to understand the soil microbiome dynamics [6]. The LTE “nutrient depletion trial” of the Justus-Liebig University of Giessen (Germany) was established in 1954 to test the effect of different fertilization regimes, namely mineral N, P, K (in different amounts and combinations) and manure (cattle) on the biomass yields of sugar beet, winter wheat and summer barley cultivated successively. Soil physico-chemical parameters and crop yields were regularly monitored during the years. However, no information is available on the response of the soil microbiome to the different fertilization regimes in this LTE.

Microorganisms play a crucial role in soil as main regulators of all biogeochemical cycles [7,8]. Soil biogeochemical cycles can also influence each other [9,10]; for example, it was demonstrated that both P and N deficiency affected P-cycling in the subsoil of the Giessen long-term fertilization experiment [11]. The influence of microorganisms on the soil nutrient turnover can eventually affect crop stability and productivity [12]. Moreover, the direct and indirect interactions between plants and microbes will also affect plant health and growth [13,14], for example by mobilization of soil nutrients [15]. Among all macro- and micro-nutrients, nitrogen is one of the most important and limiting factors for plant growth [16,17]. The nitrogen cycle is regulated by several functional categories of microorganisms, mainly prokaryotes, which include nitrifiers, denitrifiers and nitrogen fixers [18]. These functional categories of microbes on both terrestrial and marine ecosystems is driving the global nitrogen cycle [19,20].

Long-term experiments are suitable frameworks to study the response of the soil microbiome to differential soil management and fertilization practices, in terms of diversity, structure and activity. Based on molecular fingerprinting methods, it was shown that different fertilization regimes can modify the structure [21,22] and the diversity [23] of the soil bacterial communities. For example, it was shown that the amendment with organic manure increased both abundance and diversity of potential ammonia oxidizing bacterial taxa [24,25]. Usually, in such studies, only one soil habitat was examined. Due to plant growth and root activity, different soil habitats (such as rhizosphere and bulk soil) are cyclically generated during the successive crop rotations; the microbiome of these different soil habitats are expected to be influenced by the plant at different extent (more in rhizosphere and less in bulk soil).

The aim of this work was to analyze the response of the soil bacterial microbiota to different long-term nitrogen fertilization regimes (mineral N, manure and both) in the LTE nutrient depletion trial of Giessen in two soil habitats, rhizosphere and bulk soil. We hypothesized that, due to the variable extent of plant influence, the soil microbiota will show different responses to the fertilization treatments in the soil habitats. Using soil physico-chemical analysis, high-throughput DNA sequencing and quantitative PCR, we tested the effects of the different nitrogen fertilization regimes on the microbiota composition, diversity and structure, on the abundance of nitrogen cycle-related genes, and on the co-occurrence correlations within the soil microbiota.

## 2. Materials and Methods 

### 2.1. Sampling Site, Experimental Design and Sample Collection

The sampling site was the experimental station “Weilburger Grenze” of the Justus-Liebig University of Giessen, in Germany (Lat: 50.6024, Lon: 8.6540), where a nutrient depletion trial as a long-term fertilization experiment is running since 1954 [1] with the following crops: 1) sugar beet, 2) winter wheat and 3) summer barley, on a three-years rotation basis (one crop each year). The soil is classified as Fluvic Gleyic Cambisol (IUSS Working Group WRB, 2015) consisting of 6% sand, 58% silk and 36% clay in Ap horizon (0–24 cm). The nutrient fertilization treatments were 100% mineral N (indicated as “N”), 100% manure (indicated as “M”), 100% mineral-N + 100% manure (indicated as “MN”) and no nitrogen fertilization (indicated as “0”). Besides the differential nitrogen fertilization, all plants received 100% of both phosphate and potassium mineral fertilization (Table S1). One hundred percent of fertilizers corresponded to 60 kg N ha^−1^, 90 kg P ha^−1^ and 120 kg K ha^−1^ for barley and wheat; 100 kg N ha^−1^, 90 kg P ha^−1^ and 90 kg K ha^−1^ for sugar beet. The types of mineral fertilizers applied were N, lime ammonium nitrate (27% N); P, triple superphosphate (46% P_2_O_5_); K: granule potassium (60% K_2_O).

The experimental design was a randomized complete block design with four blocks, each block including one plot (4 × 4.5 m) per treatment. The four plots, representing the treatment replicates, were indicated in the sample coding as consecutive numbers (one to four).

The sampling campaign took place on September 8 th, 2015; rhizospheric and bulk soil samples were collected during the growth of sugar beet (rhizospheric soil was the soil firmly attached to the small roots emerging from the beet, while bulk soil was collected underneath the beet). For each plot, soil sampling was realized in four positions along a diagonal transect; these four subsamples of each plot were pooled into one representative sample. In total, 32 samples were analyzed (4 treatments × 2 soil habitats × 4 replicates). Soil samples were collected in sterile and DNA-free tubes, then brought to the laboratory in a cooling box and immediately sieved using sterile 2 mm sieves. Sieved soil aliquots for DNA-extraction and chemical analysis were frozen at −20 °C until DNA extraction. Soil water content was determined directly. For the statistical analyses, the factors “Fertilization treatment” (hereafter “Treatment”) and “Soil habitat” were considered; the main effect of the factors “mineral-N” and “Manure” was also tested. “Block” was included as random factor.

### 2.2. DNA Extraction and IonTorrent Sequencing

Metagenomic DNA was extracted from freshly thawed ~0.3 g of soil, using the NucleoSpin Soil Kit (Machery-Nagel, Dürren, Germany), with the extraction buffer SL2 and the enhancing buffer SX according the manufacturer guidelines. The extracted DNA was eluted in 60 µl elution buffer and the quality was controlled by 1% w/v agarose gel electrophorese stained with GelRed (Biotium, Fremont, USA). For sequencing with the Ion PGM Sequencer (Thermo Fischer Scientific, Waltham, USA), a first PCR was performed with universal primers 520F (5′-AYTGGGYDTAAAGNG-3′) [26] and 907R comp (5′-CCGTCAATTCMTTTRAGTTT-3′) [27] as described by Ambika Manirajan et al. [28]. A second PCR (five cycles) was carried in a total volume of 50 µL using the same forward and reverse primer pair but with the specific barcode sequences and an ion-adapter (Primer A key) on the forward primer and an another ion-adapter (Primer P1 key) on the reverse primer. All primers were purchased by IDT (Leuven, Belgium) and PCR conditions were the same as described by Ambika Manirajan et al. [28]. The PCR products were cleaned by using the PCR clean-up gel extraction kit (NucleoSpin Gel and PCR Clean-up, Machery-Nagel) followed by the NucleoMag NGS Clean-up and Size Selection Kit (Machery-Nagel) according to the manufacturer’s instructions. The clean PCR products were quantified with the Helixyte^TM^ Green Fluorimetric dsDNA Quantification Kit (AAT Bioquest, Sunnyvale, USA) in a TECAN GENios FL fluorescence reader (Tecan Group Ltd, Männerdorf, Switzerland) against a standard curve of known DNA concentrations. The PCR products of all samples were pooled together to an equimolar mixture, diluted to a final concentration of 26 pM and sequenced as described by Ambika Manirajan et al. [28]. Sequences were submitted to the ENA database (www.ebi.ac.uk/ena/) under the project number PRJEB27860.

### 2.3. IonTorrent Sequence Analysis

The sequences obtained from three independent IonTorrent runs were merged and analyzed with QIIME 1.9 [29]. Quality (threshold: 20) and length (200–650 nucleotides) filtering were applied. Chimeric sequences were detected using the UCHIME algorithm implemented in VSEARCH [30]. OTUs were generated at a similarity level of ≥97% using the UCLUST method [31] and the taxonomy was assigned against the SILVA 128 database [32]. Contaminant (plastidic, mitochondrial and unidentified) and singleton OTUs were deleted. To calculate the alpha- (Shannon, Phylogenetic diversity and Chao1) and beta-diversity (jackknifed-UniFrac weighted pairwise distances, [33], a normalized dataset rarefied to 4310 reads per sample was used. Beta-diversity plots (Principal Component Analysis, PCoA) were visualized by Emperor [34]. Statistical comparisons of alpha-diversity metrics were done with ANOVA and Tukey HSD Test, using SPSS version 20 (IBM Corp., Armonk, USA). The statistical model included the factors “treatment”, “soil habitat”, their interaction, and “block” as random factor. The main effect of factors “mineral-N” and “Manure” was also tested. For the beta-diversity metrics, the non-parametric multivariate analysis of variance using distance matrices (ADONIS, [35] and the analysis of similarity (ANOSIM, [36] were used, as implemented in QIIME. 

### 2.4. Co-Occurrence Network Analysis

To identify significant relationships among members of the soil microbiota and to detect stable consortia of taxa, we performed co-occurrence network analysis using the Co-occurrence Network inference software (CoNet) [37]. To reduce the compositional effect, we used not-normalized data [38]. OTUs with total abundance <1000 reads were deleted, to reduce the noise and to increase statistic robustness [38]. A distribution of pairwise scores was computed for each of the following similarity and correlation measures: Bray–Curtis, Kullback–Leibler, Pearson and Spearman. Then, we selected initial thresholds such that the initial network contained 1000 top and bottom edges. For each measure and edge, 100 permutations (with renormalization for correlation measures and row shuffling resampling) and bootstrap scores were generated. Unstable edges (outside the 2.5–97.5 percentiles of the bootstrap distribution) were removed. The single p-values generated by the four methods were then merged using the Brown’s method [39], which takes into account the dependencies between different measures. After correction for false discovery rate (FDR) using the Benjamini–Hochberg method [40], edges with merged P-values below 0.05 were kept. Considering that Bray–Curtis and Kullback–Leibler distances are both robust to compositionality, while Pearson and Spearman are both biased by compositionality but more sensitive [41], only interactions supported by three or more of these similarity measures were considered statistically very robust and thus retained.

### 2.5. Soil Chemical Analyses

For the determination of the nitrate concentrations in the two soil habitats, one gram of soil was diluted with two milliliters of deionized water, mixed in an overhead shaker (Reax 2, Heidolph Instruments, Schwalbach, Germany) and centrifuged at 16.249 × g (Mikro 20, Hettich GmbH, Tuttlingen, Germany). The supernatant was filtered through a 0.2 µm regenerated cellulose membrane (Rotilabo, Carl Roth GmbH & Co. KG, Karlsruhe, Germany) and analyzed by ion chromatography [42]. For the analyses of the ammonium concentrations in the two soil habitats, two grams of soil were extracted with 8 mL 1 M KCl, and the concentration was determined by a colorimetric assay [43]. 

pH values of soil solutions (extraction with 0.01M CaCl_2_) were measured with a pH meter (Ino Lab pH 720, WTW 82362 Weilheim, Germany).

Statistical comparisons were done with ANOVA and Tukey HSD Test, using SPSS version 20. The model included the factors “treatment”, “soil habitat”, their interaction, and “block” as a random factor. The main effect of factors “mineral-N” and “Manure” was also tested.

### 2.6. Quantitative PCR 

The quantification of functional genes involved in the nitrogen cycles and 16S rRNA genes (total bacterial abundance) was performed on bulk soil samples only. The same DNA extracts as described above were used. Quantitative PCR (qPCR) was conducted on a Rotor-Gene 3000 (Corbett Research, Sydney, Australia) by using Absolute qPCR SYBR Green Mix (ThermoFischer Scientific). The different primer pairs and the PCR conditions used to amplify the genes are listed in Table S2. Standards for the different genes were prepared from pure cultures or environmental clones as described by Kampmann et al. [44], and 10-fold serial dilutions of the standards were used as template, in triplicate, to determine the calibration curves. Total gene copy numbers of the standards were calculated according to Kampmann et al. [44]. For comparison of the abundance, the target numbers of the functional nitrogen cycles genes were expressed as relative to the total 16S rRNA gene copy number. Statistical comparisons were done with ANOVA and Tukey HSD Test, using SPSS version 20. The model included the factors “treatment”, “soil habitat”, their interaction, and “block” as random factor. 

## 3. Results

### 3.1. IonTorrent Sequencing Analysis

**Figure 1:**
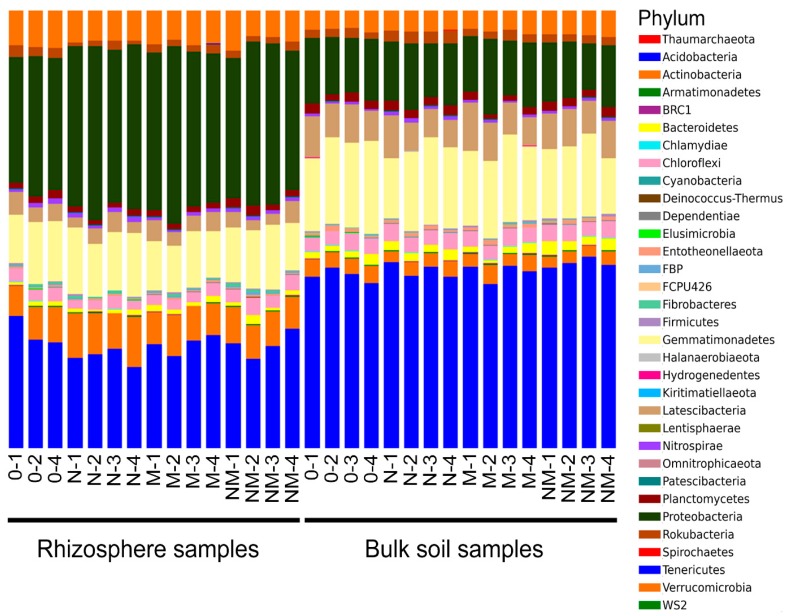
Phylum-level taxonomic composition of the bacterial microbiota in the analyzed rhizospheric and bulk soil habitats, as obtained by metabarcoding of prokaryotic 16S rRNA genes.

The sequences were produced in two different sequencing runs and were subsequently merged for the analysis. In total, 6,315,452 raw sequences were processed. After length/quality filtering and removal of chimera and contaminant sequences, 1,326,174 high-quality bacterial reads remained, which were grouped into Operational taxonomic units (OTUs) at 97% similarity level. Three thousand and fifteen OTUs had more than 10 reads and were kept for the analysis. Thirty-three prokaryotic (32 bacterial and one archaeal) phyla, 91 classes, 234 orders, 392 families, and 677 genera were identified. In rhizospheric soil, the most abundant phylum was *Proteobacteria*, while in the bulk soil *Acidobacteria* had the highest relative abundance (Figure 1). One rhizosphere sample of the treatment “0” (0–3) was removed, because it had an unreliable taxonomical composition dominated by one single OTU, and was not included into the dataset (Figure S1).

For the calculation of alpha- and beta-diversity, a rarefied dataset at a sequencing depth of 4310 reads per sample was used. The alpha-diversity metrics (diversity and richness) were significantly affected by soil habitat (ANOVA, *p* < 0.001) but not by treatment (Table 1). Furthermore, the main effects of mineral-N and manure were not significant. The interaction habitat* mineral-N was significant for the richness (*p* = 0.027) and at the border of significance for the diversity (*p* = 0.055). However, analyzing each soil separately, the Shannon index was significantly different between treatments in the rhizospheric soil (ANOVA, *p* = 0.038; Table 2).

Weighted Unifrac and Bray–Curtis distances (beta-diversity analysis) showed strong effects of the soil habitat (Adonis, *p* = 0.001) but not of the treatment (Figure S2). However, the analysis of each soil separately indicated a significant effect of the different nitrogen fertilization treatments (Table 2). This effect was very negligible in the rhizosphere, with only one OTU (representing 0.55% of the total rhizosphere microbiota) significantly affected by treatment, while it was notably greater in the bulk soil, with 66 OTUs (representing 33.1% of the total bulk soil microbiota) significantly affected by the nitrogen fertilization treatment (G-test of independence, FDR-corrected *p* < 0.05) (Table 2). These OTUs belonged to several taxa and the most represented were the phylum *Acidobacteria* with 19 OTUs and the family *Gemmatimonadaceae* with 10 OTUs (Figure S3). One of the OTUs belonged to the genera *Nitrospira*, a well-known nitrifier: it was more represented in the treatment N and reduced by manure (treatments M and NM). One OTU was identified as *Blastocatellaceae* and a second one as *Blastocatellia*; members of these taxa have not been cultured and therefore prediction of metabolisms is difficult. Interestingly, *Blastocatellia* was described as core taxa in wheat rhizosphere and additionally as hub taxa in a study on wheat rhizosphere microbiome of eight different wheat genotypes [45]. The two OTUs of *Blastocatellaceae*, found to react differently based on the nitrogen regime, showed a complementary behavior: one was more abundant and the other one reduced in the manure-amended treatments (M and NM; Figure S3). A *Bradyrhizobium* OTU was most abundant in the no-fertilization treatment and most reduced in the nitrogen-rich treatment NM (Figure S3), which is coherent with its physiology, (nitrogen fixation is in fact downregulated by high nitrogen availability). In the rhizosphere, the only OTU affected by treatment was identified as *Rhodanobacter*, a genus that includes some denitrifying species, and it was more abundant in the treatment M. 

### 3.2. Co-Occurrence Network Analysis

**Figure 2:**
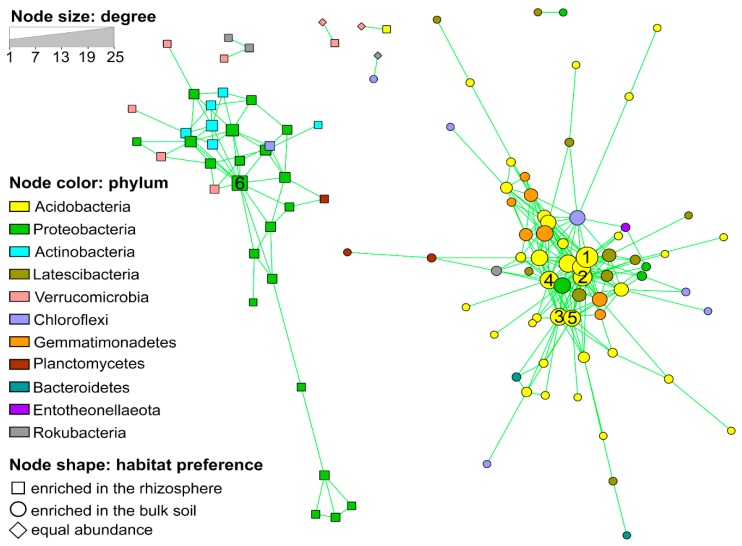
Co-occurrence network showing the positive correlations (FDR-corrected *p* < 0.05) between abundant Operational taxonomic units (OTUs) (> 0.075% of total reads) in the rhizosphere and bulk soil habitats. Circles represent OTUs, colored by taxonomy and sized by degree (number of connections). Node shapes indicate the habitat preference. Numbers indicate the hub OTUs: 1) OTU 14-Acidobacteria (Subgroup 6); 2) OTU 15-Acidobacteria (Subgroup 11); 3) OTU 28-Acidobacteria (Subgroup 6); 4) OTU 51-Acidobacteria (Subgroup 5); 5) OTU 74-Acidobacteria (Subgroup 22) and 6) OTU 22-Betaproteobacteriales (SC-I-84).

We reconstructed a co-occurrence network based on the occurrence patterns of the OTUs with >0.075% relative abundance (>1000 reads); these OTUs accounted for about 77.1% of the total microbiota. Two distinct clusters and an uncorrelated group were identified (Figure 2). Interestingly, the two clusters showed a high taxonomical coherence: Cluster 1 was characterized by the enrichment of *Proteobacteria, Actinobacteria and Verrucomicrobia*, while Cluster 2 by *Acidobacteria*, “*Latescibacteria”* and *Gemmatimonadetes*. Other bacterial groups occurred equally in both clusters (Figure 2). All OTUs of one cluster were enriched in the rhizosphere, while the OTUs of the second cluster were specific to the bulk soil. The OTUs associated to the bulk soil appeared more interconnected to each other in comparison to the rhizosphere-associated OTUs (Figure 2; Table 3). The hub taxa of the two clusters (those OTUs with highest degree, betweenness centrality and closeness centrality) were: five *Acidobacteria*–OTUs for the bulk soil cluster and one *Betaproteobacteriales*−OTU for the rhizospheric cluster (Figure 2, numbered nodes). The two hub OTUs in the bulk soil cluster identified as *Acidobacteria* Subgroup 6 (OTU 14 and 28) were also among those OTUs affected by treatment in the bulk soil, but not in the same way (Figure S3).

### 3.3. Soil Chemical Analyses

The concentration of both nitrate and ammonium was significantly affected by soil habitat (ANOVA, *p* = 0.015 and *p* = 0.039, respectively). Higher nitrate concentrations were measured in the rhizospheric soil, while higher ammonium concentrations were present in the bulk soil. However, the most significant factor was mineral-N with both nitrate and ammonium concentrations being higher in the mineral-N amended treatments (N and NM) (ANOVA, *p* < 0.001 and *p* = 0.002, respectively; see Table 4). Soil pH ranged between 6.21 and 6.58, with no significant differences between fertilization treatments (*p* = 0.46).

### 3.4. Quantitative PCR

**Figure 3:**
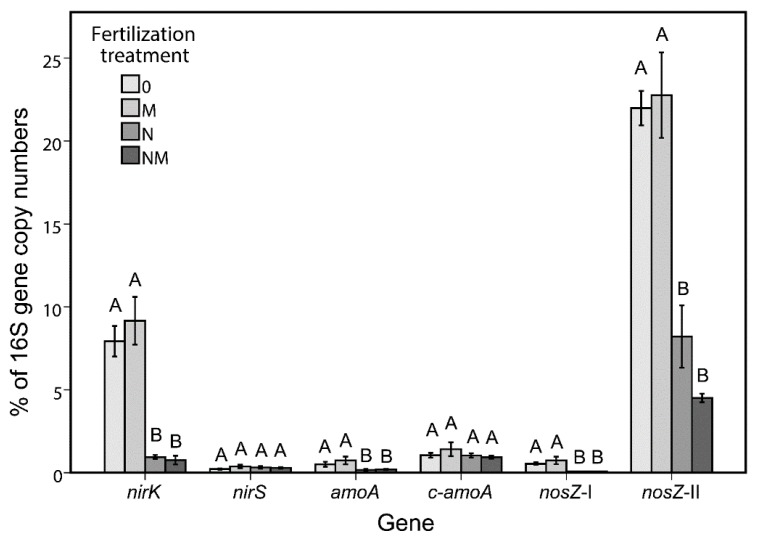
Relative abundance (% of 16S rRNA gene copy number) of nitrogen cycle-related genes (bacterial *amoA*, archaeal *amoA*, *nirK*, *nirS nosZ*-I and *nosZ*-II) in the bulk soil under the four N-fertilization treatments. Different letters above the bars indicate significantly different means (Tukey HSD, *p* < 0.05) among each gene. Treatments: 0, no N-amendment; N, mineral-N amendment; M, manure amendment; NM, mineral-N + manure amendment.

qPCR of genes related to the N-cycle in the bulk soil showed a negative effect of mineral N-fertilization on the abundance of *nirK*, *amoA*, *nosZ*-I and *nosZ*-II (Figure 3). This was observed with both absolute (number of gene copy number per gram of soil) and relative (% of 16S rRNA genes) abundances. In an attempt to identify bacterial taxa potentially involved in the nitrogen cycle, the correlation between these N-cycle genes and the OTUs was calculated for the bulk soil. In total 25 negative correlations were found with gene abundance of either *amoA, nirK, nosZ*-I or *nosZ*-II (Table 5). From the 66 OTUs affected by fertilization treatment in the bulk soil (Figure S3), 13 were negatively correlated with one or more N-cycle gene. These OTUs probably represent species that profited from the amended mineral-N and outgrew other microbes in the soil.

## 4. Discussion

In this work, we assessed the response of the bacterial soil microbiota to different N-fertilization regimes in the long-term experimental field of Giessen (Germany). We analyzed two soil habitats, rhizospheric and bulk soil, and our results indicated that the soil microbiota responded at different extents to the fertilization regimes in the two soil habitats. The changes were highest in the bulk soil whereas less in the rhizosphere, the soil habitat more influenced by the plant. This means that the working hypothesis of this study was confirmed. Neumann et al. [46] demonstrated that the response of the soil microbiome to long-term fertilization depends on soil micro-habitat; here, we extended this concept to a larger spatial scale.

We started our work with the cultivation-independent analysis of the microbiota, by high-throughput sequencing. Both alpha- and beta-diversity showed a prevailing influence of the soil habitat, and a secondary effect of the fertilization treatment; the latter was strongly different in the two soil habitats, being stronger in the bulk soil. This can be explained by the influence of the plant, which is minimal in the bulk soil compared to the rhizosphere [47,48]. Considering the bulk soil alone, most of the OTUs affected by treatment belonged to the phylum *Acidobacteria* and to the family *Gemmatimonadaceae*. Both groups are mainly composed by uncultivated members; therefore, it is hard to interpret these changes in terms of functions. Nevertheless, *Acidobacteria* were predicted to have denitrifying activity [49] and to be able to use nitrite as nitrogen source [50], which suggest their involvement in the nitrogen cycle. Instead, the metabolism of *Gemmatimonadaceae* is mostly unknown but it is known that *Gemmatimonas aurantiaca* T-27^T^ significant reduced N_2_O when oxygen was initially present [51]. Li et al. (2017) showed a correlation of *Gemmatimonadetes* with organic+inorganic fertilization. Our results confirmed a correlation of *Gemmatimonadetes* with fertilization. 

In rhizosphere, the only OTU affected by fertilization was *Rhodanobacter*, a genus that includes some denitrifying species, and it was more abundant in the treatment M. The genus *Rhodanobacter* was identified to assimilate ^13^C-labeled wheat root and potato material in a soil experiment in which ^13^C-labled DNA was analyzed for identification of the actively degrading microbiota [52,53].

Co-occurrence analysis is a useful approach to gain insights into the potential functional roles of the microbial community members, including the non-cultivated ones [8,54,55], and delivers important ecological information beside the simple alpha- and beta-diversity [56,57]. In our work, this analysis showed the existence of two clusters, each associated to one soil habitat, characterized by certain specific taxa, genes and physico-chemical parameters (Figure 2). This suggests not only a structural difference of the microbiome (to be expected from two different soil habitats) but also a segregation of functional processes. Ultimately, these differences can be linked to the redox status of the two environments. The enrichment of *Proteobacteria* in the rhizospheric soil is coherent with the higher influence of the plant: in fact, these bacterial taxa are typical members of the plant microbiome [58]. Moreover, *Gammaproteobacteria* were shown to be associated to more productive soil habitats, which was related to higher amount of available N [59,60]. In contrast, in the bulk soil, *Acidobacteria* (especially Subgroup 6) were largely enriched. Here, it clearly appeared that this group of largely unknown, yet dominant soil bacteria are involved in the nitrogen cycle (most of the treatment-affected OTUs in bulk soil belonged to this group, Figure S3). The hub taxa identified in the two network clusters represent those species with a major role in maintaining the stability of the community [61]. Interestingly, two of these “hub taxa” were significantly enriched in the NM fertilization treatment, which suggests a direct influence of the combined mineral-N + organic fertilization on key species of the nitrogen cycle, in the soil microbial network.

The second part of the work was dedicated to the analysis of the soil physico-chemical parameters and the quantification of the genes involved in the nitrogen cycle (archaeal and bacterial *amoA*, *nirS*, *nirK*, *nosZ*-I and *nosZ*-II). Nitrogen and ammonium concentrations showed significant differences between soil habitats (Table 4). In particular, more nitrate was found in the rhizospheric soil, which can be explained by the higher amount of root exudates coupled with the more aerobic conditions enabling nitrification: in fact, the bulk soil (showing less nitrate and more ammonium) was sampled deeper (under the sugar beets), thus being more compressed and anoxic. However, this analysis highlighted the effect of the mineral-N fertilization, which resulted in an increase of both nitrogen compounds in both soil habitats. This had a strong negative impact on the functional genes analyzed in the bulk soil. We chose bulk soil for the qPCR analysis of the functional genes because this soil habitat showed the strongest effect of the different fertilization regimes on the microbiota. In our study, the crenarchaeal ammonia monooxygenase gene (*c-amoA*) was ~20 times more abundant than the bacterial counterpart (Figure 3), coherently with previous results [62]. *nosZ*-II was more abundant than the *nosZ*-I. A higher abundance of *nosZ*-II compared to *nosZ*-I was also found in other soils [63,64]. Interestingly, we found abundant *Gemmatimonadaceae* in the bulk soil, where they also seems to play a role, and *Gemmatimonas* is known to have a *nosZ*-II gene [63].

## 5. Conclusions

From the results of our investigation, we can draw the following conclusions:

1. In the long-term fertilization experiment in Giessen (Germany), the different nitrogen fertilization regimes affected the soil microbiota differentially depending on the soil habitat. Thus, it should be considered that it is not possible to extrapolate general conclusions when only one soil habitat is analyzed.
2. Bulk soil was the soil habitat most affected by different nitrogen fertilizations. For future studies, we suggest using this soil habitat for investigating the effects of long-term fertilization regimes on the structure and function of the soil microbiota.
3. The soil microbiota appeared organized in strongly interconnected clusters, which were also taxonomically coherent and soil habitat-specific (habitat complementarity). The cluster enriched in the bulk soil was characterized by *Acidobacteria* and appeared more strongly interconnected.
4. *Acidobacteria* were the hub taxa in the bulk soil. These hub taxa deserve future attention, as they appear to be functional key regulators of the soil microbiome.
5. The soil microbiome acts as a single functional unit. Therefore, a better understanding of the factors driving the interactions between microbial species, and how these correlate with the physico-chemical parameters of the soil is needed to optimize soil management practices for a more sustainable agriculture. Here, we showed that, despite the strong influence of the soil habitat on the general microbiome structure and function, the N-fertilization regime (in particular, the mineral + organic) significantly influenced two of the five most important OTUs of the soil microbial network of the bulk soil.
6. Mineral-N fertilization has a strong negative impact on the functional genes related to the nitrogen cycle. However, it seems this is an indirect effect, which can be attributed to the stimulation of opportunistic microbes, which use the provided nitrogen.

Our results indicate trajectories for planning future research on long-term perspective studies of the microbiome in arable soils. Furthermore, the investigations have proven new experimental evidence enabling sustainable management of differential nitrogen fertilization methods on arable land.

## Figures and Tables

**Table 1:** Alpha-diversity metrics of the soil bacterial microbiota, calculated at Operational taxonomic unit (OTU_97_) level, in the two soil habitats, under the four N-fertilization treatments. Different letters indicate significantly different means (Tukey HSD, *p* < 0.05).

Soil habitat	Fertilization Treatment	Shannon Index (Diversity)	N. of OTUs (Richness)
Bulk soil	N	7.92 ± 0.07 BC	767.0 ± 21.7 BCD
	M	7.80 ± 0.06 C	730.3 ± 17.1 D
	NM	7.93 ± 0.07 BC	768.5 ± 15.5 BCD
	0	7.87 ± 0.07 C	746.8 ± 19.3 CD
Rhizosphere	N	8.23 ± 0.02 A	824.3 ± 3.83 ABC
	M	8.33 ± 0.06 AB	861.5 ± 16.6 A
	NM	8.34 ± 0.06 A	850.8 ± 14.7 AB
	0	8.42 ± 0.06 A	869.7 ± 21.8 A

**Table 2:** Significance *p* values of alpha- and beta-diversity metrics for the factor “fertilization treatment”, calculated on individual soil habitats, and number of OTUs significantly affected by the same factor in each individual soil habitat. Significant values (*p* < 0.05) are highlighted in bold. Phil. Div. = phylogenetic diversity.

Soil Habitat	Alpha-Diversity (ANOVA)			Beta-Diversity (Adonis)		N. of OTUs Affected by Treatment (% of Total Reads) ^1^
	Shannon	Phil. Div.	Richness	Bray–Curtis	Weighted Unifrac	
Rhizosphere	0.038	0.366	0.213	0.025	0.035	1 (0.55 %)
Bulk	0.536	0.200	0.377	0.030	0.020	66 (33.1 %)

^1^ identified by *g*-test of independence at FDR-corrected *p* < 0.05.

**Table 3:** Network parameters of the co-occurrence network shown in Figure 3.

Network Parameter	Rhizosphere Cluster	Bulk Soil Cluster
N. of nodes	33	65
Avg. N. of neighbors	4.12	6.46
Clustering coefficient	0.396	0.389
Network centralization	0.329	0.299
Network density	0.129	0.101

**Table 4:** Concentrations of nitrate and ammonium in the two soil habitats, under the four N-fertilization treatments. Different letters indicate significantly different means (Tukey HSD, *p* < 0.05). Fertilization treatments: N, mineral-N; M, manure; NM, mineral-N + manure; 0, no N-amendment.

Soil Habitat	Fertilization Treatment	Nitrate µmol NO_3_ * g soil_dw_^−1^	Ammonium µg NH_4_ –N * g soil_dw_^−1^
Bulk soil	N	71.2 ± 24.9 AB	8.38 ± 2.69 A
	M	30.6 ± 4.1 B	0.90 ± 0.06 B
	NM	66.4 ± 22.4 AB	2.82 ± 0.60 AB
	0	41.6 ± 8.8 B	0.95 ± 0.08 B
Rhizosphere	N	107.6 ± 22.2 AB	2.31 ± 0.58 AB
	M	66.5 ± 7.4 AB	0.94 ± 0.05 B
	NM	136.7 ± 15.1 A	1.95 ± 0.44 AB
	0	29.7 ± 5.7 B	0.67 ± 0.05 B

**Table 5:** Significant Spearman correlations (at FDR-corrected *p*< 0.05) between relative abundances of bacterial Operational taxonomic units (OTUs) and qPCR values of the N-cycle genes affected by fertilization treatments (see Figure 3), in the bulk soil.

Gene	N. of Positively Correlated OTUs	N. of Negatively Correlated OTUs
*amoA*	0	1
*nirK*	0	11
*nosZ*-I	0	4
*nosZ*-II	0	9

## Supplementary Materials

The following are available online at https://www.mdpi.com/2073-4425/11/4/456/s1, Figure S1: UPGMA tree of beta-diversity distances, Figure S2: Beta-diversity plot of the soil bacterial microbiota, calculated at OTU_97_ level, in the two soil habitats, under the four N-fertilization treatments, Figure S3: OTUs significantly affected by fertilization treatment in the bulk soil, Table S1: Details of the fertilization treatments, Table S2: Thermal profiles and primers used for qPCR.
