# Supplementary material for: The Response of the Soil Microbiota to Long-Term Mineral and Organic Nitrogen Fertilization is Stronger in the Bulk Soil than in the Rhizosphere
Source: Genes (Basel). 2020 Apr 22;11(4):456. doi: 10.3390/genes11040456 (PMC7230438; doi:10.3390/genes11040456)
Supplement: Supplementary file 1 [file genes-11-00456-s001.pdf]

Supplementary information to:

The response of the soil microbiota to long term mineral and organic nitrogen fertilization is stronger in the bulk soil than in the rhizosphere

Massimiliano Cardinale, Stefan Ratering, Aitak Sadeghi, Sushil Pokhrel, Bernd Honermeier, Sylvia Schnell

- Figure S1: UPGMA tree of beta-diversity distances
- Figure S2: Beta-diversity plot of the soil bacterial microbiota, calculated at OTU_{97} level, in the two soil habitats, under the four N-fertilization treatments
- Figure S3: OTUs significantly affected by fertilization treatment in the bulk soil
- Table S1: Details of the fertilization treatments
- Table S2: Thermal profiles and primers used for qPCR
- Additional bibliography to Tab. S2

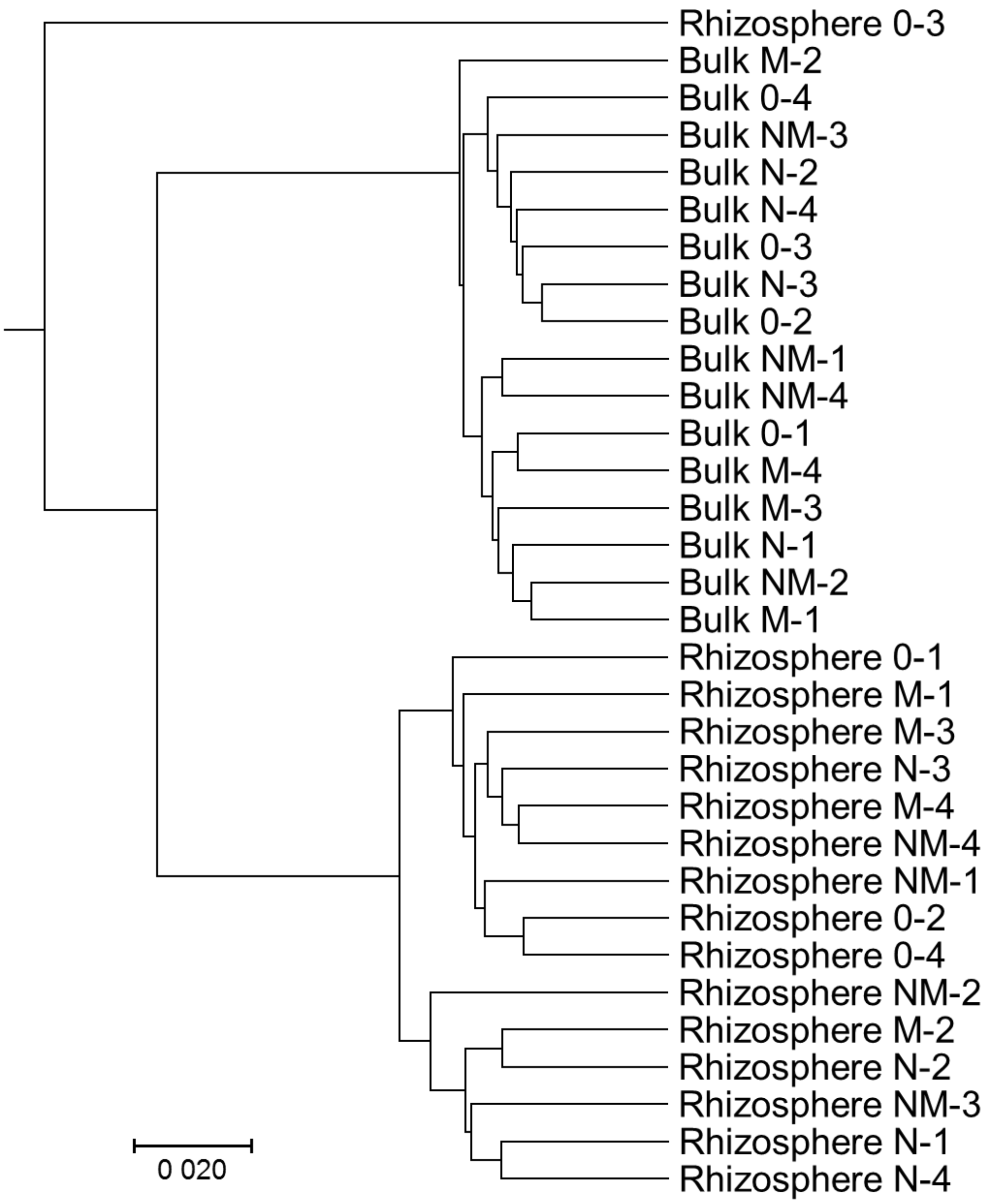

Figure. S1 UPGMA tree of beta-diversity distances. UPGMA tree showing the similarity of the taxonomic structure (weighted Unifrac distances) between the microbiotas of the analyzed soil samples (at OTU_{97} level). The sample "Rhizosphere 0–3" was removed from the analysis because it clearly did not cluster within the universe of the data.

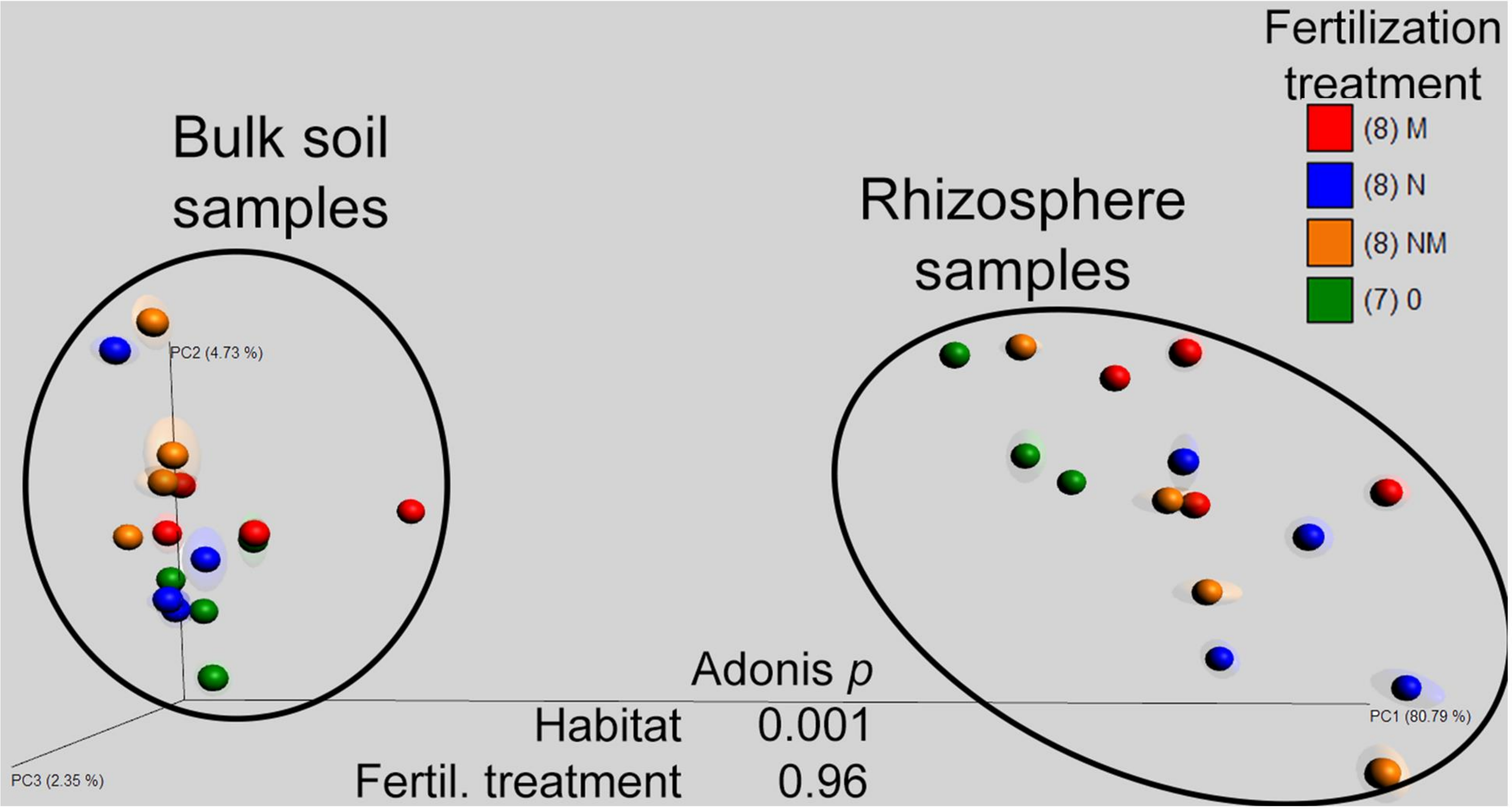

Figure S2. Beta-diversity plot of the soil bacterial microbiota, calculated at OTU_{97} level, in the two soil habitats, under the four N-fertilization treatments. Plot is based on weighted Unifrac distances, showing the similarity of the microbiota structure of all soil samples. Adonis *p* values for the factors "habitat" and "fertilization treatment" are indicated. Fertilization treatments: N, mineral-N; M, manure; NM, mineral-N + manure; 0, no N-amendment.

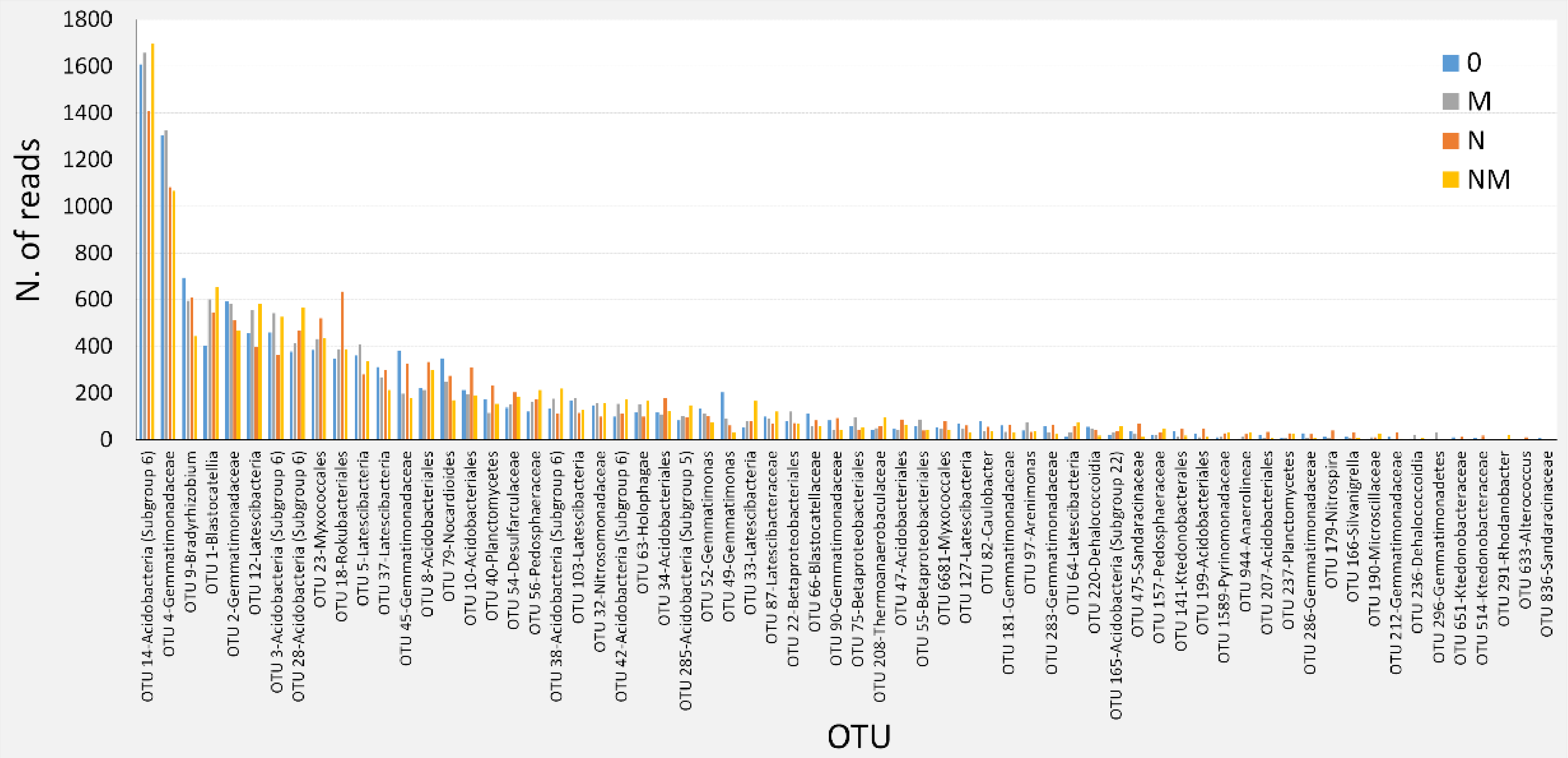

Figure. S3 OTUs significantly affected by fertilization treatment in the bulk soil. Significance was assessed by g-test of independence (FDR-corrected *p* < 0.05). Treatments: 0, no N-amendment; N, mineral-N amendment; M, manure amendment; NM, mineral-N + manure amendment.

Table S1 Details of the fertilization treatments. The types of mineral fertilizers applied were: Mineral-N: lime ammonium nitrate (27% N); Phosphate: triple superphosphate (46% P_2O_5); Potassium: granule potassium (60% K_2O)

Treatment name	Nutrients			
	Mineral-N	Manure	Phosphate	Potassium
N	100%	0%	100%	100%
M	0%	100%	100%	100%
NM	100%	100%	100%	100%
0	0%	0%	100%	100%

Table S2 Thermal profiles and primers used for qPCR. All qPCRs reactions started with an initial denaturation step at 95 °C for 15 min and ended with a final elongation step at 72 °C for 3 min. For the acquisition of the fluorescence signal an addition step of 80 °C were performed after the elongation step.

Target gene	Primer names	Primer [µM]	BSA [µg $µl^{-1}$]	Program	Cycles	Source standard DNA	Literature
16S rRNA	520F<br>926R comp	0.8/0.6	0.2	95 °C/45 s, 55 °C/45 s, 72 °C/60 s	40	Environmental clone	Claesson et al., 2009<br>Engelbrektson et al., 2010
nirk	nirk876<br>nirk5R	0.25/0.5	0.5	95 °C/20 s, 63 °C/25 s, 72 °C/20 s	40	*Bradyrhizobium japonicum*	Henry et al., 2004<br>Braker et al., 1998
nirS	cd3af<br>R3cd	0.25/0.5	0.5	95°C/20 s, 63 °C/25 s, 72 °C/20 s	40	*Cupriavidus necator*	Throbäck et al., 2004
Bacterial *amoA*	amoA1F<br>amoA2R	0.25/0.25	0.2	95 °C/30 s, 59 °C/30 s, 72°C/ 20 s	35	Environmental clone	Rotthauwe et al., 1997
Archaeal *amoA*	CamoA-19F<br>CamoA-616R	0.25/0.5	0.5	95 °C/30 s, 64 °C/45 s, 72 °C/45 s	40	Environmental clone	Pester et al., 2012
nosZ-I	nosZ2F<br>nosZ2R	0.25/0.25	-	95 °C/20 s, 63 °C/25 s, 72 °C/20 s	40	*Pseudomonas fluorescens*	Henry et al., 2006
nosZ-II	nosZ-II<br>nosZ-II-R	0.25/0.5	-	95 °C/30 s, 63 °C/50 s, 72 °C/50 s	40	Environmental clone	Jones, 2013

Additional bibliography for Table S2

Braker, G., Fesefeldt, A., Witzel, K.P., 1998. Development of PCR primer systems for amplification of nitrite reductase genes (*nirK* and *nirS*) to detect denitrifying bacteria in environmental samples. Appl. Environ. Microbiol. 64 (10), 3769–3775.

Henry, S., Baudoin, E., López-Gultiérrez, J.C., Martin-Laurent, F., Brauman, A., Philippot, L., 2004. Quantification of denitrifying bacteria in soils by *nirK* gene targeted real-time PCR. J. Microbiol. Meth. 59, 327–335. Corrigendum.

Henry, S., Bru, D., Stres, B., Hallet, S., Philippot, L., 2006. Quantitative detection of the nosZ gene, encoding nitrous oxide reductase, and comparison of the abundances of 16S rRNA, *narG*, *nirK*, and *nosZ* genes in soils. Appl. Environ. Microbiol. 72 (8), 5181–5189.

Jones, C.M., Graf, D.R.H., Bru, D., Philippot, L., Hallin, S., 2013. The unaccounted yet abundant nitrous oxide-reducing microbial community: A potential nitrous oxide sink. ISME J. 7 (2), 417–426. 10.1038/ismej.2012.125.

Pester, M., Rattei, T., Flechl, S., Gröngröft, A., Richter, A., Overmann, J., Reinhold-Hurek, B., Loy, A., Wagner, M., 2012. *amoA*-based consensus phylogeny of ammonia-oxidizing archaea and deep sequencing of *amoA* genes from soils of four different geographic regions. Environ. Microbiol. 14 (2), 525–539.

Rotthauwe, J.H., Witzel, K.P., Liesack, W., 1997. The ammonia monooxygenase structural gene *amoA* as a functional marker: Molecular fine-scale analysis of natural ammonia-oxidizing populations. Appl. Environ. Microbiol. 63 (12), 4704–4712.

Throbäck, I.N., Enwall, K., Jarvis, A., Hallin, S., 2004. Reassessing PCR primers targeting *nirS*, *nirK* and *nosZ* genes for community surveys of denitrifying bacteria with DGGE. FEMS Microbiol. Ecol. 49, 401–417.
